# Tactile Routing for Location Privacy Preservation in Wireless Sensor Networks: A Game Theoretic Approach

**DOI:** 10.3390/s22197334

**Published:** 2022-09-27

**Authors:** Mujahid N. Syed, Uthman Baroudi

**Affiliations:** 1 Interdisciplinary Research Center for Intelligent and Secure Systems, Department of Industrial Systems Engineering, King Fahd University of Petroleum & Minerals, Dhahran 31261, Saudi Arabia; 2 Interdisciplinary Research Center for Smart Cities and Logistic, Department of Computer Engineering, King Fahd University of Petroleum & Minerals, Dhahran 31261, Saudi Arabia

**Keywords:** wireless sensor networks, location privacy preservation, game theory, incomplete information in payoffs

## Abstract

Location Privacy Preservation (LPP) in Wireless Sensor Networks (WSNs) during the era of the Internet of things and smart systems is a critical element in the success of WSNs. LPP in WSN can be stated as: given a WSN with an adversary aiming to unravel the location of critical nodes of a WSN, the goal of the WSN manager is to enshroud the location of the critical nodes via routing and/or encryption mechanisms. Typical research in the LPP of WSN routing involves developing and/or estimating the performance of a fixed routing protocol under a given attack mechanism. Motivated by advancements in network softwarization, in this work, we propose an approach where the WSN manager as well as the WSN adversary can deploy multiple routing and attack mechanisms, respectively. Initially, the proposed approach is formulated as a repeated two-player zero-sum game. The formulation is further extended to handle multiple objectives and incomplete information in the game matrix. In this work, the multiple objectives are handled via the epsilon constraint method. The presence of incomplete information in the formulation is modeled as interval based uncertainty. To sum, the proposed formulation ultimately boils down to linear programming problems, which can be efficiently solved. Numerical case studies to showcase the applicability of the proposed approach are illustrated in this work. Finally, discussion on obtaining the required data from any given WSN, discussion and interpretation of the formulation’s results, and future research direction of the current work is presented.

## 1. Introduction

The Internet of things (IoT) is the heart of Industry 4.0, where a number of smart devices are deployed on a communication network. The devices process the incoming and outgoing data ubiquitously, and they communicate among themselves autonomously. Thus, IoT is the crux in developing new applications and experiences including smart factories, smart agriculture, smart homes, etc. The smart devices of IoT play key roles in collecting, processing, and forwarding data from sources nodes to the sink node(s) via multiple hop network communication. This ubiquitous and autonomous communication results in Wireless Sensor Networks (WSNs) with new challenges that are related to their sustainability. On one hand, since these devices are of constrained capabilities such as energy supply, transmission, range, computing power, etc., lots of research works focus on developing routing methods that cope with these devices. On the other hand, these networks are typically deployed and operated in unattended environments. Therefore, these networks might be vulnerable to various type of attacks that may impact the operation of the network partially or completely, such as identifying the location of critical network nodes (such as source(s) and sink(s) nodes). Securing the location of critical nodes in WSNs is a complicated, pressing challenge that needs to be tackled on different fronts.

The seminal work presented in [1] highlights the importance of the Location Privacy Preservation (LPP) of critical nodes in a WSN. Specifically, the authors depicted, via an example of Panda–Hunter Game, the importance of LPP. For example, a habitat containing pandas is monitored via many wireless sensor nodes that are randomly distributed across the habitat. The sensor (source) nodes monitor and send information about pandas to a base (sink) node. The information received at the base station can be used for the well-being of pandas in the habitat. On the other hand, a hunter is interested in identifying the location of pandas, which can be indirectly achieved by identifying the location of the source nodes. Thus, the hunter moves into the habitat and monitors the network traffic. Tracing the network communication packets (even without actually looking inside the packet) can reveal the location of the source nodes, thereby compromising the location of pandas. Thus, LPP routing methods are critical along with the packet encryption methods.

In this work, we focus on improving routing methods under the assumption that the adversary (generalized term for the hunter in the above example) lacks the ability to decipher the encrypted packets. The conventional research on LPP in WSNs typically assumes a specific intrusion model, and the primary goal is to develop a corresponding routing method that is better in terms of location privacy. In addition to that, the secondary goals may include: reduced age of information and energy consumption. One of the naive assumptions in the conventional approaches is the prepossession of a specific intrusion model. Since the intrusion can be executed by one or more adversaries, surviving one or more intrusion techniques with one specific routing method may not be fruitful in preserving the location of the critical nodes over the network’s time horizon.

Motivated by the advent of network softwarization and advancements in the Soft Defined Network (SDN) and Network Functions Virtualization (NFV) [2,3], our goal is to propose and develop a hybrid routing model where the network communications are carried out via multiple routing mechanisms. Such a tactile routing model provides the means to handle multiple intrusions via multiple techniques. Later, we discuss the applicability of this approach in real networks.

The rest of the paper is organized as follows. Section 2 presents the relevant literature on WSN routing and attack mechanisms. The problem statement is established in Section 3. A connection between the proposed LPP problem and game theory is highlighted in Section 4. Section 5 illustrates relevant experiments, and provides discussion on the experiment data and results. Section 6 concludes the paper.

## 2. WSN Routing and Attack Mechanism

The LPP for WSNs first appeared in [1]. With the development of IIoTs and extensive applicability of WSNs, the topic of LPP has received widespread attention in the recent decade. Well-known routing ideas available in the LPP literature are categorized as:

**Random Walk Routing:** In this routing, packets are sent from source node to sink node over random walks or paths [4,5]. This is one of the simplest forms of routing, where the sensor nodes do not calculate or store much information. However, this routing often leads to high overall energy consumption due to longer source-to-sink path length [6]. Typically, random walk routing is deployed with additional protocols. For example, see [7,8,9], which involves random walk routing with other routing methods.
**Phantom Routing:** In this routing, packets are sent from source node to sink node in two phases [1,10]. In the first phase, the packets are sent from source node to phantom node (a node in the network that is typically neither a source nor a sink). In the second phase, the packets are sent from the phantom node to sink node. Typically, in the first phase, random walk routing is used, and in the second phase, shortest path routing is used. The usage of multiple phantom nodes is proposed in [11]. The key difficulty of phantom routing is the selection of phantom nodes. A technique based on sector domain to increase positional diversity of phantom nodes is proposed in [12].
**Fake Packets Routing:** In this routing, along with the real packets, the network is induced with fake packets. The idea is to camouflage real packets among the fake packets. The key difficulty in this routing is the right number of fake packets to be induced into the network. Furthermore, fake packets consume network resources, including energy and time. Applications of fake packets can be seen in [13,14].
**Ring Routing:** The idea in this routing (see [15]) is to induce disjoint rings (cycles) while transmitting the packets from source nodes to sink node(s). Every disjoint ring contains a master node (bus node) that is responsible for forwarding the packets from source node to sink node (actual route). Simultaneously, the bus nodes on the actual route send additional packets to a ring of nodes. This additional set of packets sent on the disjoint rings confuses the adversaries. The further applications of ring routing can be seen in [16], and energy efficient ring routing can be seen in [17].
**Multi-Path Routing:** In multi-path routing, data between source and sink nodes are sent via multiple disjoint paths. In order to avoid tracing back of the paths by the adversary, some deviation mechanisms are proposed. For example, a random stride routing is proposed in [18], and a constrained random routing is proposed in [19]. The constrained routing limits the energy usage in the network. Typically, multi-path routing without careful deployment suffers from high energy consumption during the transmission. Thus, several flavors of this category focusing on reduced energy consumption in multi-path routing are proposed in [20,21,22].
**Network Encoded Routing:** In this routing, packets are re-encoded, split into multiple small packets, or integrated into one big packet during the transmission.
**Directional Routing:** Directional routing consists of directional antennas and/or transceivers. The underlying assumption is that the directional routing makes eavesdropping expensive. In [23], the usage of directional antennas along with transmitting power control and information compression is depicted. More usage of directional transceivers is presented in [24].
**Data Mule Routing:** Data mules are nothing but mobile agents. The routing mechanism is as follows: a source node sends packet to a randomly selected intermediate node. The intermediate node forwards the packet to a data mule. The data mule randomly moves around the network and forwards the packet to another intermediate node. Finally, the intermediate node forwards the packet to the sink node. The data mule makes it difficult for adversaries to locate the critical nodes, but it increases the transmission time.
**Context-Aware Routing:** The idea here is to use sensor nodes that can detect and localize the adversary. Such a mechanism can then be used for local temporary shutdown (stop working for a while) of nodes around the adversary [25,26]. This shutdown acts as an entrapment of the adversary. Moreover, based on the distance of adversary from the source or sink nodes, alternate routes avoiding the adversary’s coverage area can be used.

## 3. Problem Statement

Consider a Wireless Sensor Network (WSN) where an adversary makes passive attacks to identify (or locate) the critical nodes. The adversary is free to choose any attack mechanisms, including, for example:

- Eavesdropping.
- Tracing Back.
- Traffic Analysis.
- Node Compromised.
- ID Analysis.
- Collusion Stacks.

Let *A* be the set of attacking mechanisms available with the adversary and a∈A be an attack mechanism. It is assumed that the adversary is capable of launching local or hotspot-level attacks on the WSN. The goal of the WSN manager is to maximize the safety period, while ensuring the Quality of Service (QoS) with reasonable energy consumption. In this work, we assume that the WSN is designed such that multiple routing mechanisms can adaptively be employed, so the WSN manager (defender) could command the nodes in a WSN to choose among these different routing mechanisms. For example, the WSN manager (defender) could choose any of the following routing mechanisms for a certain period, and then choose another one for the next period, and so on according to a specific defense strategy:

- Random Walk.
- Ring Routing.
- Phantom Base Station.
- Fake Packet Injection.
- Multi-path Routing.
- Data Mule.
- Hiding Mechanism.
- Directional Communication.
- Isolation Mechanism.

Let *D* be the set of options available with the defender and d∈D be a defense mechanism. Let $h_{d,a}$ be the total survival time (the time from the start of attacks to the detection of critical station(s)) when the adversary picks an attack mechanism *a* and the defender picks a defense mechanism *d*. The primary goal of the defender is to have high survival time, which is contrary to the goal of the adversary. Therefore, the payoff for the adversary conflicts with the defender and can be taken as $-h_{d,a}$. Thus, the conflict between adversary and defender can be modeled as a two-player zero sum-game. However, unlike the conventional game theory models, the manager has to satisfy the QoS and energy requirements. In addition to that, the data of survival time may contain uncertainty. All the notations that are used in this paper are defined in Abbreviations. In the following section, a game-theory-based approach to LPP is proposed.

## 4. Modeling WSN LPP as a Matrix Game

The relation between network designers/administrators and intruders/attackers can be modeled as a non-cooperative game. Hence, security- and privacy-based protocols in computer networks can be designed using game theory. Game-theory-based approaches related to privacy in Internet-based communications can be seen in works [27,28,29,30,31,32]. In this work, a strategic game between the WSN manager (as the defender) and the WSN attacker (as the adversary) is proposed. The roles of the defender and the adversary, under mild assumptions, can be cast as a two-player zero-sum game. The following are the key assumptions and characteristics of the proposed game:

- Rational players: Both players have well-defined individual goals, and they strive to reach their goals.
- Non-cooperative players: Both players are competitive, and their individual goals are diametrically opposed.
- Finite moves: Both players have a finite set of moves to play. A move for the defender (adversary) can be defined as a selection of a routing (counterattack) mechanism.
- Independent moves: Unlike typical two-player games, in this game, there are no fixed time points where the players play their moves. Practically, the players can play the moves asynchronously and at different frequencies. However, the moves are taken independently, i.e., no player knows the choice of the other player a priori.
- Memory-less repeated moves: The game is a multiple-shot game, where the moves of the defender are not at all visible to the adversary (and vice versa), even after the end of the game. Thus, it exhibits memory-less nature, where none of the players learn from the other based on the past shots or moves.
- Expected preference: For every combination of defender move and attacker move, a payoff is known or can be estimated. This payoff captures the preference relation for each player. Both players prefer to obtain the best of the expected payoff value.

Thus, due to the above nature of the game, it can be approximated as a steady-state, two-player zero-sum game [33]. Since the moves are taken by the players over and over again during the network’s operation, it is advisable for the defender to use different pure strategies on each move. Therefore, the optimal mixed strategy obtained from the two-player zero-sum game can be utilized as tactical operational probabilities for the defender.

### 4.1. Deterministic Survival Times

Let $H\in R^{D\times A}$ be a matrix, where the *d*th row and *a*th column element $h_{d,a}$ represents the survival time when an adversary picks an attack mechanism a∈A and a defender picks a defense mechanism d∈D. Let us assume that $h_{d,a}$ is crisp and deterministic. The expected payoff *E* of the game, gain for the defender (loss for the adversary), is given as follows:

(1) $$E=x^{T}\mathrm{Hy}$$

where $x={\left[x_{1},\ldots ,x_{\left|D\right|}\right]}^{T}$, $x_{d}$ is the probability of using the strategy *d* by the defender, $y={\left[y_{1},\ldots ,y_{\left|A\right|}\right]}^{T}$, and $y_{a}$ is the probability of using the strategy *a* by the adversary. In the case of mixed strategies, the objective of the defender is to maximize the minimum expected payoff, whereas the objective of the adversary is to minimize the maximum expected payoff.

**Lemma** **1.** *The Operations Research (OR) model to identify the best mixed strategy for the defender (also known as max-minimizer) can be written as:*

(2) $$\underset{x\geq 0}{\max:}\min_{a\in A}\left\{h_{a}^{T}x|e^{T}x=1\right\}$$

*where $h_{a}$ is the ath column of matrix H, e represents a vector containing all ones, and its size can be identified from the context.*

**Proof1.** Let $E_{d}\left(x\right)$ be the worst expected payoff value of the defender for any arbitrary choice of x, defined as:

(3) $$E_{d}\left(x\right)=\min_{\begin{array}{c} e^{T}y=1,\\ y\geq 0 \end{array}}\left\{x^{T}\mathrm{Hy}\right\}$$

The worse expected value can be re-written as:

(4a) $$\begin{array}{cc} E_{d}\left(x\right)= & \min_{\begin{array}{c} e^{T}y=1,\\ y\geq 0 \end{array}}\left\{x^{T}\mathrm{Hy}\right\} \end{array}$$

(4b) $$\begin{array}{cc} \;\;\;\;\;\;= & \min_{\begin{array}{c} e^{T}y=1,\\ y\geq 0 \end{array}}\left\{\sum_{a\in A}{\left(H^{T}x\right)}_{a}y_{a}\right\} \end{array}$$

(4c) $$\begin{array}{cc} \;\;\;= & \min_{a\in A}\left\{{\left(H^{T}x\right)}_{a}\right\} \end{array}$$

(4d) $$\begin{array}{cc} \;\;= & \min_{a\in A}\left\{h_{a}^{T}x\right\} \end{array}$$

where ${\left(H^{T}x\right)}_{a}$ is the *a*th element of vector $H^{T}x$. Equation (4c) follows from the theory of linear programming, which states that the optimal solution of a linear function $\sum_{a\in A}{\left(H^{T}x\right)}_{a}y_{a}$ on a non-empty simplex $\left\{e^{T}y=1,y\geq 0\right\}$ lies at one of the |A| extreme points. Thus, the OR model that provides the best mixed strategy for the defender (also known as max-minimizer) can be written as:

(5) $$\underset{\begin{array}{c} e^{T}x=1,\\ x\geq 0 \end{array}}{\max:}\min_{a\in A}\left\{h_{a}^{T}x\right\}$$

The above formulation is the same as Formulation (2). □

**Corollary** **1.** *The OR model to identify the best mixed strategy for the attacker can be written as:*

(6) $$\underset{y\geq 0}{\min:}\max_{d\in D}\left\{h_{d}^{T}y|e^{T}y=1\right\}$$

*where $h_{d}$ is the dth row of matrix H.*

**Lemma** **2.** *The proposed game has a mixed strategy Nash equilibrium.*

**Proof2.** Based on the linear programming’s weak duality theory [34], the following holds:

(7) $$\min_{a\in A}\left\{h_{a}^{T}x|e^{T}x=1,\;x\geq 0\right\}\;\leq \;\max_{d\in D}\left\{h_{d}^{T}y|e^{T}y=1,y\geq 0\right\}$$

Notice that both Formulations (2) and (6) are feasible. From the linear programming’s strong duality theory, it can be concluded that the optimal objective values for both formulations are equal and finite, say *v*. That is, the following holds:

(8) $$\underset{x\geq 0}{\max:}\min_{a\in A}\left\{h_{a}^{T}x|e^{T}x=1\right\}\;=\;\underset{y\geq 0}{\min:}\max_{d\in D}\left\{h_{d}^{T}y|e^{T}y=1\right\}\;=\;v$$

Let $x^{*}$ and $y^{*}$ be defined as follows:

(9) $$\begin{array}{cc} x^{*}= & \;\underset{x\geq 0}{argmax}\left\{\min_{a\in A}\left\{h_{a}^{T}x|e^{T}x=1\right\}\right\} \end{array}$$

(10) $$\begin{array}{cc} y^{*}= & \;\underset{y\geq 0}{argmin}\left\{\max_{d\in D}\left\{h_{d}^{T}y|e^{T}y=1\right\}\right\} \end{array}$$

Equation (8) indicates that, no matter what the adversary (defender) chooses to play, the defender (adversary) has no rational to change from $x^{*}$ ($y^{*}$). That is, neither can hope to improve the expected payoff value of $x^{*}T{\mathrm{Hy}}^{*}$. Thus, ($x^{*}$, $y^{*}$) represents the mixed strategy Nash equilibrium of the proposed game.

In practical scenarios, the defender (adversary) may have additional objectives that are unrelated to the adversary (defender). For example, the defender might be inclined to deviate from the optimal mixed strategy in order to reduce energy consumption and/or improve QoS in communication. Formulation (2) can be extrapolated to include the QoS and energy objectives. Incorporation of the additional objectives for the defender’s model may invalidate the above minimax equality. However, the resulting strategy will ensure that QoS and energy consumption in the communication network are at the preferred level. A Multi-Objective Linear Programming (MOLP) model that captures optimal mixed strategy for the defender can be written as:

$$\begin{array}{cc} max.:\; & \end{array}$$

(11a) $$\begin{array}{cc} \; & \left\{\lambda ,-q^{T}x,-p^{T}x\right\}\;\;\;\;\;\\ \;\;\;\;\;\;\;\;\;s.t.: & \end{array}$$

(11b) $$\begin{array}{cc} \; & \;\;\;\;\;\lambda \leq h_{a}^{T}x\;a\in A \end{array}$$

(11c) $$\begin{array}{cc} \; & \;\;e^{T}x=1 \end{array}$$

(11d) $$\begin{array}{cc} \; & \;x\geq 0 \end{array}$$

where the QoS level expressed in terms of the transmission latency, obtained by strategy *d*, is represented by $q_{d}$ for all d∈D, and $q={\left[q_{1},\ldots ,q_{\left|D\right|}\right]}^{T}$. Similarly, the energy consumption for strategy *d* is represented by $p_{d}$ for all d∈D, and $p={\left[p_{1},\ldots ,p_{\left|D\right|}\right]}^{T}$. Constraints (11c) and (11d) ensure that x results in the probability of selecting the defense strategy. Constraint (11b) along with the first objective in Function (11a) maximizes the worst case survival time. The other two objective functions correspond to transmission latency and energy minimization.

Let $p_{max}=p^{T}x^{*}$ and $q_{max}=q^{T}x^{*}$. Formulation (11a–d) can be equivalently solved as a series of the following OR models, where $\varepsilon_{1}$ and $\varepsilon_{2}$ are parameters that are selected a priori:

$$\begin{array}{cc} max.: & \end{array}$$

(12a) $$\begin{array}{cc} \; & \lambda \\ s.t.: & \end{array}$$

(12b) $$\begin{array}{cc} \; & \;\;\;\;\;\;\lambda \leq h_{a}^{T}x\;a\in A \end{array}$$

(12c) $$\begin{array}{cc} \; & \;\;e^{T}x=1 \end{array}$$

(12d) $$\begin{array}{cc} \; & \;\;\;\;\;q^{T}x\leq q_{max}-\varepsilon_{1} \end{array}$$

(12e) $$\begin{array}{cc} \; & \;\;\;\;\;p^{T}x\leq p_{max}-\varepsilon_{2} \end{array}$$

(12f) $$\begin{array}{cc} \; & \;\;x\geq 0 \end{array}$$

When $\varepsilon_{1}=\varepsilon_{2}=0$, the solution of Formulation (12a–f) results in minimax game value. However, when $\varepsilon_{1}\neq 0$ or $\varepsilon_{2}\neq 0$, then the solution deviates from the minimax game value. Obtaining the solution for various values of $\varepsilon_{1}$ and $\varepsilon_{2}$ results in a Pareto surface, which can be presented to the WSN manager. The network manager and decision makers can then select the operational point from the Pareto. In the following subsection, we extend the proposed model to handle uncertainty in the H matrix.

### 4.2. Incomplete Information of Survival Times

Uncertainty in H matrix is typically referred as incomplete information in payoffs. Both probabilistic and non-probabilistic methods that handle uncertainty in payoffs are available in the literature [35,36,37,38,39]. Uncertain payoffs in the form of interval-valued payoffs [40,41,42,43] are related to the proposed WSN game.

In this work, we assume that the uncertainty in estimating parameter H exists. Furthermore, we assume that the uncertainty can be represented by a bounded interval. Without loss of generality, let the interval based payoff matrix of defender be expressed as $\Omega ={\left(\omega \right)}_{d,a}=\left(\left[{\underline{h}}_{d,a},{\bar{h}}_{d,a}\right]\right)$, where ${\left(\omega \right)}_{d,a}$ is a closed interval, ${\underline{h}}_{d,a}$ is the lower bound on the closed interval, and ${\bar{h}}_{d,a}$ is the upper bound of the closed interval.

Note that the above uncertainty keeps the zero-sum nature of the game intact, since the payoff matrix of the attacker will be nothing but $-\Omega ={\left(-\omega \right)}_{d,a}=\left(\left[-{\bar{h}}_{d,a},-{\underline{h}}_{d,a}\right]\right)$. Similar to the methodology presented in [41] for handling interval-based uncertainty, we transform Formulation (12a–f) into two formulations: one for obtaining lower bound (pessimistic game) and another for obtaining upper bound (optimistic game). The formulations are as follows:

$$\begin{array}{cc} max: & \end{array}$$

(13a) $$\begin{array}{cc} \; & \bar{\lambda }\\ s.t.: & \end{array}$$

(13b) $$\begin{array}{cc} \; & \;\;\;\;\;\;\;\;\;\bar{\lambda }\leq {\bar{h}}_{a}^{T}\bar{x}\;a=1,\ldots ,A \end{array}$$

(13c) $$\begin{array}{cc} \; & \;\;\;e^{T}\bar{x}=1 \end{array}$$

(13d) $$\begin{array}{cc} \; & \;\;\;\;\;\;q^{T}\bar{x}\leq {\bar{q}}_{max}-\varepsilon_{1} \end{array}$$

(13e) $$\begin{array}{cc} \; & \;\;\;\;\;\;p^{T}\bar{x}\leq {\bar{p}}_{max}-\varepsilon_{2} \end{array}$$

(13f) $$\begin{array}{cc} \; & \;\;\bar{x}\geq 0 \end{array}$$

$$\begin{array}{cc} max: & \end{array}$$

(14a) $$\begin{array}{cc} \; & \underline{\lambda }\\ s.t.: & \end{array}$$

(14b) $$\begin{array}{cc} \; & \;\;\;\;\;\;\;\;\;\underline{\lambda }\leq {\underline{h}}_{a}^{T}\underline{x}\;a=1,\ldots ,A \end{array}$$

(14c) $$\begin{array}{cc} \; & \;\;\;e^{T}\underline{x}=1 \end{array}$$

(14d) $$\begin{array}{cc} \; & \;\;\;\;\;\;q^{T}\underline{x}\leq {\underline{q}}_{max}-\varepsilon_{1} \end{array}$$

(14e) $$\begin{array}{cc} \; & \;\;\;\;\;\;p^{T}\underline{x}\leq {\underline{p}}_{max}-\varepsilon_{2} \end{array}$$

(14f) $$\begin{array}{cc} \; & \;\;\underline{x}\geq 0 \end{array}$$

where the values of $\left({\bar{q}}_{max},{\bar{p}}_{max}\right)$ and $\left({\underline{q}}_{max},{\underline{p}}_{max}\right)$ are obtained using (9) by replacing $h_{a}^{T}$ with ${\bar{h}}_{a}$ and ${\underline{h}}_{a}$, respectively. Upon solving Formulations (13a–f) and (14a–f), we obtain the upper bound $\bar{\lambda }$ and the lower bound $\underline{\lambda }$, respectively, for a given value of $\varepsilon_{1}$ and $\varepsilon_{2}$. To obtain the Pareto surfaces, a grid search on different values of $\varepsilon_{1}$ and $\varepsilon_{2}$ is conducted. The solution of the above two models results in two Pareto surfaces. The network manager and/or the decision maker can pick a desired operational point from the Pareto surfaces.

## 5. Numerical Experiments and Discussion

In this section, three case studies are presented to illustrate the usage of the proposed approach. Case Study-1 is a toy example that highlights the notion of mixed strategy, steady state analysis, and data estimation. In Case Study-2, synthetic data on an arbitrary 6×6 game are utilized to depict the applicability of the proposed game. In Case Study-3, a non-square game with overlapping intervals, which is most likely related to the real-world scenarios, is presented.

### 5.1. Case Study-1

As a toy example, let us consider the case where a defender can pick between Phantom Routing (PR) and Multi-path Routing (MR). On the other hand, the adversary can pick between Traffic Analysis (TA) and Tracing Back (TB). From the literature, the following payoff matrix (illustrated in Table 1) can be constructed. Furthermore, the average values of energy and latency presented in Table 2 can be attributed to the above routing mechanisms.

Clearly, the 2×2 game presented in Table 1 has no *pure strategy* Nash equilibrium. In fact, this game is similar to the famous *Matching Pennies* game [47], which is a cornerstone example for the notion of mixed strategy Nash equilibrium and the concept of steady state analysis of the games. Indeed, Table 1 and Table 2 are not constructed from a comprehensive literature survey, and the studies in the literature may involve other parameters that may result in biased comparisons. Nevertheless, the sole purpose of Case Study-1 is to establish the following points:

- H,p,q can be estimated beforehand from either simulation or emulation study of the actual networks.
- Mixed strategy Nash equilibrium is strongly applicable to the proposed defender and adversary game.

### 5.2. Case Study-2

The survival time payoff matrix H containing the interval-based normalized survival times (values between (0, 1]) are given in Table 3. The normalized values in the range [0.2,0.4] may indicate short (small) survival time, latency or energy consumption, [0.5,0.7] indicates medium survival time, latency or energy consumption, and [0.8,1] indicates long survival time, latency or energy consumption. For the routing mechanisms, Table 4 presents the energy p and the latency q values. Suitable units for survival time, energy, and latency can be obtained from the actual network. A discussion on obtaining the above data from actual WSN is presented at the end of this section. The case study has the following key structure: The defender can change the routing policy from time to time. The defender can pick from any six routing mechanisms, say: R1,R2,…R6. On the other hand, the adversary can change the attacking mechanism from time to time. The adversary can pick from any six attack mechanisms, say: A1,A2,…A6.

**Figure 1:**
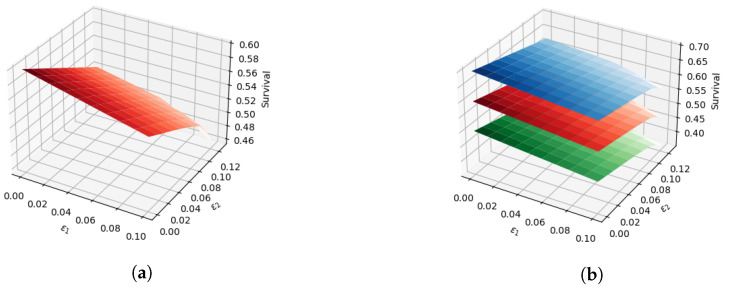
Case Study-2, Pareto surfaces. (**a**) Formulation (12a–f) Pareto. (**b**) Formulations (12a–f)–(14a–f) Pareto surfaces.

The grid for $\varepsilon_{1}$ is generated by taking 10 equally spaced intervals of $\left[0,0.2\times q_{max}\right]$, $\left[0,0.2\times {\bar{q}}_{max}\right]$, and $\left[0,0.2\times {\underline{q}}_{max}\right]$ for solving Formulations (12a–f)–(14a–f), respectively. Similarly, for $\varepsilon_{2}$, the grid is generated by taking 10 equally spaced intervals of $\left[0,0.2\times p_{max}\right]$, $\left[0,0.2\times {\bar{p}}_{max}\right]$, and $\left[0,0.2\times {\underline{p}}_{max}\right]$ for solving Formulations (12a–f)–(14a–f), respectively. All the models are solved using the GLPK solver (https://www.gnu.org/software/glpk/, accessed on 1 September 2022 ). The Pareto surface obtained after solving Formulation (12a–f) is depicted in Figure 1a, and the Pareto surfaces obtained after solving Formulations (13a–f) and (14a–f) are illustrated in blue and green colored surfaces in Figure 1b, respectively. Table 5 displays the results of the case study at $\varepsilon_{1}=\varepsilon_{2}=0$, which indicates the minimax game values. From Table 5, it can be concluded that if the defender ignores the latency- and energy-related objectives, then playing R1,R2 or R6 equally will likely be the best for LPP. Practically, it means that the network administrator can alternate between R1,R2 or R6 to obtain the longest possible survival time, while preserving the privacy location of the sink node. Other operational points can be extracted from Figure 1b.

### 5.3. Case Study-3

**Figure 2:**
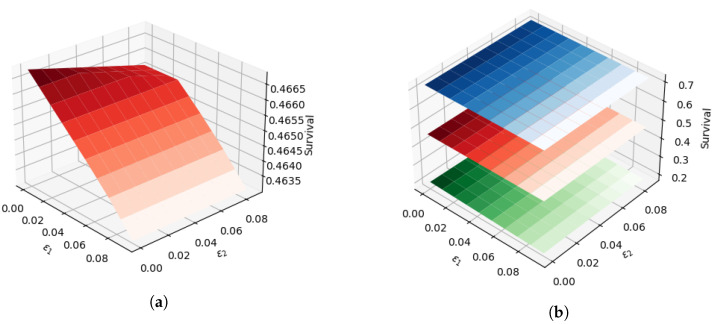
Case Study-3, Pareto surfaces. (**a**) Formulation (12a–f) Pareto. (**b**) Formulations (12a–f)–(14a–f) Pareto surfaces.

Case Study-3 is similar to the setup of Case Study-2. The key difference is in the data. The interval widths in Case Study-2 are constant, and the intervals are non-overlapping. In this case study, the interval widths are not constant, and the intervals are overlapping for a given row or column. The data for Case Study-3 are presented as follows: The survival time payoff matrix H containing the interval-based survival times are given in Table 6, and Table 7 presents the energy p and the latency q values. All the models are solved using the open source GLPK solver. The Pareto surface obtained after solving Formulation (12a–f) is depicted in Figure 2a, and the Pareto surfaces obtained after solving Formulations (13a–f) and (14a–f) are illustrated in blue and green colored surfaces in Figure 2b, respectively. Table 8 displays the results of the case study at $\varepsilon_{1}=\varepsilon_{2}=0$, which indicates the minimax game values. From Table 8, it can be concluded that if the defender ignores the latency- and the energy-related objectives, then, under optimistic circumstances, playing R3,R5,R6,R7 or R8 with the given probabilities in Table 8 is the best for LPP. In practice, this could mean that R7 is deployed 36.9% of the time, R3 is deployed 26.3% of the time, R8 is deployed 14.4% of the time, and so on. Similarly, under pessimistic circumstances, playing R1,R2,R4,R5,R6 or R7 with the given probabilities in Table 8 is the best for LPP.

### 5.4. Estimating H,p,q

In order to implement the proposed technique, WSNs and IOTs should have flexible network design such that they can employ different routing protocols alternatively during their operations. The advancements in network softwarization could facilitate the existence of such networks. In [48], the authors surveyed the softwarization of Unmanned Aerial Vehicle (UAV) routing, while [49] presented the existing literature on AI-enabled routing protocols for UAV networks. Other applications of softwarization include, but are not limited to, Routing algorithm optimization in software-defined network Wide Area Network (WAN) [50], integrating Multi-path TCP (MPTCP), and Segment Routing (SR) paradigms over SDN/NFV [51].

Once such networks are established, H,p,q can be easily obtained either by developing a simulation model or extracting empirical data from emulated networks. The simulation and emulation models should have the ability to estimate the survival times, latency, and energy for a pair of routing and attack mechanisms. Thus, for a given network under consideration, the data H,p,q should be estimated via simulation model a priori by the WSN manager or the decision maker. A parametric estimation of H,p,q can be a future research direction.

## 6. Conclusions

In this work, we present a seed for a novel direction towards designing a multi-routing Wireless Sensor Network. This approach can be argued by the fact that the adversary can choose and/or change attacking mechanisms at any given time during the time horizon. Thus, the WSN manager should have the ability to choose/alternate among different routing protocols for the longevity of the network’s LPP. Furthermore, the game theoretic model provides the WSN manager with a guide on proportion of time dedicated to each routing protocol, under optimistic, average, and pessimistic circumstances. We believe the usage of multiple routing protocols with suggested time proportions will be very effective on Location Privacy Preservation (LPP). In the future, we plan to investigate the challenges of implementing this approach in real networks and study the impact of other possible payoffs on the network performance, such as computational complexity, bandwidth utilization, and memory consumption.

## Figures and Tables

**Table 1:** Survival time payoffs for the defender 2×2 game.

	TA	TB
PR	Long [44]	Medium [1,10,45]
MR	Medium [21]	Long [46]

**Table 2:** Energy (p) and Latency (q) for each routing mechanism of the 2×2 game.

	PR	MR
**p **	High	Medium
**q**	Long	Medium

**Table 3:** Survival time payoffs for the defender in Case Study-2.

	A1	A2	A3	A4	A5	A6
**R1**	[0.5, 0.7]	[0.8, 1]	[0.5, 0.7]	[0.8, 1]	[0.2, 0.4]	[0.2, 0.4]
**R2**	[0.2, 0.4]	[0.5, 0.7]	[0.2, 0.4]	[0.5, 0.7]	[0.8, 1]	[0.8, 1]
**R3**	[0.5, 0.7]	[0.8, 1]	[0.8, 1]	[0.2, 0.4]	[0.5, 0.7]	[0.2, 0.4]
**R4**	[0.8, 1]	[0.2, 0.4]	[0.2, 0.4]	[0.5, 0.7]	[0.8, 1]	[0.5, 0.7]
**R5**	[0.2, 0.4]	[0.5, 0.7]	[0.5, 0.7]	[0.8, 1]	[0.2, 0.4]	[0.8, 1]
**R6**	[0.8, 1]	[0.2, 0.4]	[0.8, 1]	[0.2, 0.4]	[0.5, 0.7]	[0.5, 0.7]

**Table 4:** Energy (p) and Latency (q) for each routing mechanism in Case Study-2.

	R1	R2	R3	R4	R5	R6
**p**	0.9	0.7	0.2	0.6	0.5	0.3
**q**	0.4	0.6	0.7	0.3	0.8	0.5

**Table 5:** Minimax game values (at $\varepsilon_{1}=\varepsilon_{2}=0$) of Case Study-2.

	Optimistic	Average	Pessimistic
**R1**	0.333	0.333	0.333
**R2**	0.333	0.333	0.333
**R3**	0	0	0
**R4**	0	0	0
**R5**	0	0	0
**R6**	0.333	0.333	0.333

**Table 6:** Survival time payoffs for the defender in Case Study-3.

	A1	A2	A3	A4	A5
**R1**	[0.38, 0.83]	[0.23, 0.63]	[0.33, 0.59]	[0.09, 0.74]	[0.39, 0.99]
**R2**	[0.36, 0.81]	[0.24, 0.79]	[0.0, 0.78]	[0.39, 0.74]	[0.25, 0.5]
**R3**	[0.16, 0.91]	[0.28, 0.73]	[0.13, 0.73]	[0.0, 0.85]	[0.16, 0.56]
**R4**	[0.0, 0.61]	[0.2, 0.85]	[0.36, 0.76]	[0.33, 0.59]	[0.0, 0.59]
**R5**	[0.09, 0.95]	[0.2, 0.7]	[0.21, 0.51]	[0.30, 0.8]	[0.4, 0.8]
**R6**	[0.09, 0.55]	[0.01, 0.91]	[0.25, 0.5]	[0.42, 0.52]	[0.10, 0.7]
**R7**	[0.11, 0.96]	[0.02, 0.62]	[0.16, 0.76]	[0.09, 0.75]	[0.38, 0.88]
**R8**	[0.0, 0.59]	[0.0, 0.89]	[0.47, 0.97]	[0.04, 0.5]	[0.33, 0.58]

**Table 7:** Energy (p) and Latency (q) for each routing mechanism in Case Study-3.

	R1	R2	R3	R4	R5
**p**	0.8	0.3	0.5	0.5	0.4
**q**	0.7	0.5	0.4	0.2	1

**Table 8:** Minimax game values for Case Study-3 (at $\varepsilon_{1}=\varepsilon_{2}=0$).

	Optimistic	Average	Pessimistic
**R1**	0	0.227	0.152
**R2**	0	0	0.351
**R3**	0.263	0	0
**R4**	0	0	0.299
**R5**	0.13	0.196	0.12
**R6**	0.094	0	0.074
**R7**	0.369	0.577	0.003
**R8**	0.144	0	0

## Data Availability

Not applicable.

## Abbreviations

*D*	a set of defense mechanisms available with the defender
*A*	a set of attack mechanisms available with the adversary
*d*	a defense mechanism, where d∈D
*a*	an attack mechanism, where a∈A
$h_{d,a}$	the average survival time when the adversary picks an attack mechanism
	*a* and the defender picks a defense mechanism *d*
${\bar{h}}_{d,a}$	the upper bound or optimistic value of $h_{d,a}$
${\underline{h}}_{d,a}$	the lower bound or pessimistic value of $h_{d,a}$
H	payoff matrix of survival times, where the *d*th row and *a*th column element
	is $h_{d,a}$
Ω	uncertain payoff matrix of survival times, where the *d*th row and *a*th column
	element is $\omega_{d,a}=\left[{\underline{h}}_{d,a},{\bar{h}}_{d,a}\right]$
$h_{a}$	the *a*th a column of matrix H
$h_{d}$	the *d*th row of matrix H
${\bar{h}}_{a}$	a vector containing the optimistic values from the *a*th column of matrix Ω
${\underline{h}}_{a}$	a vector containing the pessimistic values from the *a*th column of matrix Ω
*E*	the expected payoff of the game, gain for the defender (loss for the adversary)
$E_{d}\left(x\right)$	the worst expected payoff value of the defender for any arbitrary choice of x
$x_{d}$	the probability of using the mechanism *d* by the defender under average
	uncertainty conditions
x	vector representing probabilities of using different defense mechanisms
	under average uncertainty conditions, defined as $x={\left[x_{1},\ldots ,x_{\left|D\right|}\right]}^{T}$
$y_{a}$	the probability of using the mechanism *a* by the adversary under average
	uncertainty conditions
y	a vector representing probabilities of using different attack mechanisms
	under average uncertainty conditions, defined as $y={\left[y_{1},\ldots ,y_{\left|A\right|}\right]}^{T}$
$\bar{x}$	a vector representing probabilities of using different defense mechanisms
	under optimistic uncertainty conditions
$\underline{x}$	a vector representing probabilities of using different defense mechanisms
	under pessimistic uncertainty conditions
$x^{*}$, ${\bar{x}}^{*}$, and ${\underline{x}}^{*}$	the optimal value of x, $\bar{x}$, and $\underline{x}$, respectively
$y^{*}$	the optimal value of y
$q_{d}$	average transmission latency that can be obtained during the execution of
	defense mechanism *d*
$p_{d}$	average amount of energy that is required for implementing defense
	mechanism *d*
q	a vector representing transmission latency for all defense mechanisms
	$q={\left[q_{1},\ldots ,q_{\left|D\right|}\right]}^{T}$
p	a vector representing energy consumption for all defense mechanisms
	$p={\left[p_{1},\ldots ,p_{\left|D\right|}\right]}^{T}$
$q_{max}$	the expected transmission latency at operating point $x^{*}$, defined as
	$q_{max}=q^{T}x^{*}$
$p_{max}$	the expected energy consumption at operating point $x^{*}$, defined as
	$p_{max}=p^{T}x^{*}$
${\bar{p}}_{max}$ and ${\underline{p}}_{max}$	the expected energy consumption at operating points ${\bar{x}}^{*}$ and ${\underline{x}}^{*}$, respectively,
	defined as ${\bar{p}}_{max}=p^{T}{\bar{x}}^{*}$ and ${\underline{p}}_{max}=p^{T}{\underline{x}}^{*}$
${\bar{q}}_{max}$ and ${\underline{q}}_{max}$	the expected transmission latency at operating points ${\bar{x}}^{*}$ and ${\underline{x}}^{*}$, respectively,
	defined as ${\bar{q}}_{max}=q^{T}{\bar{x}}^{*}$ and ${\underline{q}}_{max}=q^{T}{\underline{x}}^{*}$
λ, $\bar{\lambda }$, and $\underline{\lambda }$	the worst payoff for the defender during average, optimistic, and pessimistic
	uncertainty conditions, respectively
e	a vector containing all ones
$\varepsilon_{1}$ and $\varepsilon_{2}$	parameters for epsilon constraint method
